# Numerical analysis of the pressure drop across highly-eccentric coronary stenoses: application to the calculation of the fractional flow reserve

**DOI:** 10.1186/s12938-018-0503-7

**Published:** 2018-05-30

**Authors:** R. Agujetas, M. R. González-Fernández, J. M. Nogales-Asensio, J. M. Montanero

**Affiliations:** 1 Depto. de Ingeniería Mecánica, Energética y de los Materiales and Instituto de Computación Científica Avanzada (ICCAEx), Universidad de Extremadura, Avda. de Elvas s/n, 06006 Badajoz, Spain; 2 Servicio de Cardiología, Hospital Infanta Cristina, Avda. de Elvas s/n, 06006 Badajoz, Spain

**Keywords:** CFD, Stenosis, Fractional flow reserve

## Abstract

**Background:**

Fractional flow reverse (FFR) is the gold standard assessment of the hemodynamic significance of coronary stenoses. However, it requires the catheterization of the coronary artery to determine the pressure waveforms proximal and distal to the stenosis. On the contrary, computational fluid dynamics enables the calculation of the FFR value from relatively non-invasive computed tomography angiography (CTA).

**Methods:**

We analyze the flow across idealized highly-eccentric coronary stenoses by solving the Navier–Stokes equations. We examine the influence of several aspects (approximations) of the simulation method on the calculation of the FFR value. We study the effects on the FFR value of errors made in the segmentation of clinical images. For this purpose, we compare the FFR value for the nominal geometry with that calculated for other shapes that slightly deviate from that geometry. This analysis is conducted for a range of stenosis severities and different inlet velocity and pressure waveforms.

**Results and conclusions:**

The errors made in assuming a uniform velocity profile in front of the stenosis, as well as those due to the Newtonian and laminar approximations, are negligible for stenosis severities leading to FFR values around the threshold 0.8. The limited resolution of the stenosis geometry reconstruction is the major source of error when predicting the FFR value. Both systematic errors in the contour detection of just 1-pixel size in the CTA images and a low-quality representation of the stenosis surface (coarse faceted geometry) may yield wrong outcomes of the FFR assessment for an important set of eccentric stenoses. On the contrary, the spatial resolution of images acquired with optical coherence tomography may be sufficient to ensure accurate predictions for the FFR value.

## Background

A stenosis is an abnormal narrowing in a blood vessel. Coronary arteries are known to be common sites for stenoses, which frequently lead to myocardial infarction and even sudden cardiac death. The vast majority of these stenoses are caused by atheromatous lesions, which give rise to highly eccentric narrowing. They are frequently characterized by a lumen lying in the outer region of the coronary artery, and delimited by an arc of nearly normal wall [1].

Coronary revascularization is performed to treat stenoses with hemodynamic significance. The decision to carry out this procedure is frequently based on the stenosis severity, defined as the percentage of narrowing of the coronary artery. Thus, coronary revascularization is often conducted for severe stenoses, i.e., those with severities above the threshold value 75%. While the stenosis severity can be measured with relatively non-invasive computed tomography angiography (CTA), there is no simple relationship between that parameter and the stenosis hemodynamic significance and the plaque vulnerability [2]. In fact, more than 50% of the stenoses judged severe by CTA do not cause ischemia [3]. Therefore, criteria based on measured stenosis hemodynamic significance seem to be more appropriate.

Fractional flow reverse (FFR) is defined as the ratio of maximal (i.e., on maximum hyperaemia conditions) coronary blood flow through a stenotic artery to the maximal blood flow in the hypothetical case that the artery were normal. Because this definition is not operational, it is frequently substituted by $\text{FFR}\equiv \overline{P}_1/\overline{P}_0,$ where $\overline{P}_0$ and $\overline{P}_1$ are the averages over the cardiac cycle of the pressure waveforms $P_0(t)$ and $P_1(t)$ proximal and distal to the stenosis, respectively, both measured at maximum hyperaemia. Assuming that the loss of pressure in a normal epicardial artery is negligible as compared to that in the coronary microcirculation, the two definitions would be equivalent if the distal pressure were directly proportional to the coronary blood flow [4]. This last condition does not strictly hold due to the colateral flow, among other factors, as indicated by the non-zero pressure intercept for flow in the coronary circulation [5]. In any case, the above fraction is adopted as the operational definition of FFR.

**Fig. 1:**
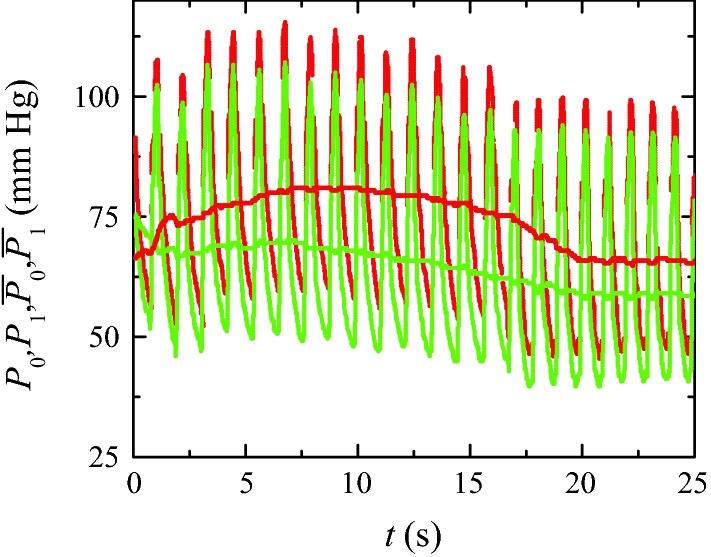
Pre-stenotic $P_0(t)$ (red lines) and post-stenotic $P_1(t)$ (green lines) pressure waveforms over 25 cycles. The FFR value 0.83 is taken as the minimum of the ratio between the functions $\overline{P}_0(t)$ and $\overline{P}_1(t)$ (smooth curves) calculated from those waveforms. The pressure waveforms were provided by the *Hospital Infanta Cristina* in Badajoz (Spain)

In practice, the pre-stenotic and post-stenotic pressure waveforms are measured over several cardiac cycles. The functions

1 $$\begin{aligned} \overline{P}_i(t)=\int _t^{t+\tau } P_i(t') \ dt' \quad i=0\;\text{and}\;1\end{aligned}$$

are calculated from those waveforms, where $\tau$ is the mean cardiac cycle period. The FFR value is taken as the minimum of the ratio $\overline{P}_1(t)/\overline{P}_0(t)$ between those two functions (Fig. 1).

FFR is the gold standard assessment of the hemodynamic significance of coronary stenoses. Revascularization is typically conducted for lesions with FFR < 0.8 [5]. FFR-guided revascularization has proved to be long-lived and cost-saving [6]. As compared with other assessments, such as angiography for multivessel evaluation (AME), FFR-guided revascularization exhibits lower rates of major adverse cardiac events. In addition, it correctly classifies as hemodynamically insignificant many lesions judged “obstructive” by AME [7]. The main drawback of the FFR-based assessment is its invasive character. In fact, this method uses pressure wires to determine the pressure waveforms proximal and distal to the stenosis, which requires the catheterization of the coronary arteries. Less invasive methods to determine the FFR value of coronary stenoses are very desirable.

Computational fluid dynamics (CFD) is a method with high diagnostic performance to determine coronary lesions that cause ischemia [8–13]. In particular, it enables the calculation of the FFR value from relatively non-invasive measurements [10, 11, 14]. CFD has been frequently used to analyze idealized 2D (axisymmetric) stenoses and understand relevant aspects of hemodynamics in these geometries [15–20], especially turbulence. There are much fewer studies dealing with 3D (asymmetric) shapes, most of them restricted to small eccentricities [21–23]. As mentioned above, many of the coronary stenoses exhibit highly eccentric shapes whose necks are delimited by an arc of the normal artery wall and a atheromatous lesion. Analyzing the influence of different hemodynamics aspects and model approximations on the pressure loss across these stenoses is of great importance, and has practical consequences in calculating the FFR value.

As mentioned above, FFR is measured at maximum hyperaemia, which greatly reduces the artery expansion/contraction during the systole/diastole phase. Therefore, one can safely adopt the rigid wall approximation in the CFD simulations, which avoids modelling the fluid solid interaction [24, 25]. FFR is a measure of the mechanical energy dissipated by viscosity in the stenosis averaged over the cardiac cycle. In some phases of this cycle, the blood speed decreases and so does the shear rate over the entire fluid domain. Therefore, it is natural to wonder whether using the Newtonian approximation with the viscosity at infinite shear rate is a good approximation to get reliable predictions for the FFR value. Here, we will answer this question by calculating the pressure drop across a stenosis with both the Newtonian and Carreau models.

It is well known that, due to the stabilizing effect of the blood acceleration, turbulence in arteries arises at Reynolds numbers much larger than those for steady flow. The peak Reynolds number in a coronary stenosis is typically much smaller than that leading to the laminar-to-turbulent transition in an oscillatory flow inside a cylindrical duct, and consequently the flow is usually assumed to be laminar. However, blood greatly accelerates in severe stenoses (like the ones studied here), which gives rise to complex post-stenotic flows, including separation, recirculation, as well as localized transition to turbulence and relaminarization [15]. Both the inlet flow perturbations and the stenosis asymmetry enhance the post-stenotic transition to turbulence [21], and thus considerable turbulent outbreaks can appear in that region even for inlet Reynolds numbers as low as 500 [17, 26]. CFD simulations of the coronary system are conducted in the laminar regime because most of the flow is truly laminar, and turbulence (if there is any) is restricted to the post-stenotic region. However, the flow in this very region essentially determines the FFR value, and therefore significant errors may be made in neglecting turbulence. Here, we will evaluate the influence of turbulence on the pressure drop across the stenosis by using the large eddy simulation (LES) model, which has shown to provide good results for asymmetric shapes [21].

Typically, the only (if there is any) available kinematic information at the stenosis inlet section is the instantaneous flow rate crossing that section [27, 28]. To complete the velocity inlet boundary condition, one frequently assumes the uniform velocity profile compatible with the measured flow rate. In fact, the pulsatility of the flow in arteries hinders the inwards viscous diffusion of momentum from the wall, which explains why the uniform velocity approximation is commonly preferred. However, significant extra loss of pressure may occur in coronary stenoses due to the growth of the boundary layer when that approximation is made [29]. In this work, we will examine the influence of this choice on the pressure drop across the stenosis.

Simulating the entire cardiac cycle increases very considerably the computing time. It implies running the simulation over several cycles to ensure that the periodic regime has been reached. The Womersley number (the ratio between local acceleration and viscous force per unit mass) takes relatively low values in the epicardial arteries. One may wonder whether the flow pulsatile character can be neglected in calculating the FFR value. In that case, the unsteady simulation could be replaced with a stationary one with an average (effective) inlet velocity. Here, we will assess to what extent the instantaneous pressure drop across the stenosis is affected by the blood acceleration.

Ideally, the FFR quantification should be conducted by simulating the flow in the stenotic region exclusively. This would allow one to increase the spatial resolution of the simulation, capturing the critical influence of the intricate stenosis shape on the FFR value. However, such a simulation requires the precise knowledge of the boundary conditions in the pre- and post-stenotic segments, which cannot be easily determined. For this reason, the numerical simulation normally involves not only the coronary artery where the stenosis is located, but also the peripheral vasculature, including the aorta. The inlet and outlet boundary conditions for this numerical domain are relatively well established, which allows CFD to produce FFR predictions in an autonomous way, i.e., just from the knowledge of the vasculature geometry and some patient-specific parameters.

The above mentioned approach does not lack important disadvantages. Even if the boundary conditions are correctly prescribed [30–32], the disparity between the sizes of the coronary stenosis and the whole numerical domain constitutes an important barrier to obtain accurate results. It is obvious that the pressure drop across the stenosis critically depends on the complicated shape of the latter. The vasculature geometrical reconstruction has limited accuracy. The stenosis shape is determined from segmentation of CTA images of artery sections. This segmentation is carried out by manually selecting a range of values in the Hounsfield scale corresponding to the area enclosed by the artery inner wall. Pixels that have been excluded (included) erroneously in this process are added (eliminated) also manually. The contours are the boundaries that separate the selected pixels from the rest. Finally, the detected contours are used to construct a 3D representation of the real vasculature geometry in terms of triangles connected to each other (the faceted geometry). One can identify at least two sources of errors in this process. Firstly, the contour detection produces errors in the artery wall position at least on the order of 1-pixel size. Lastly, both hardware and software characteristics limit the total number of triangles used in the geometrical representation of the stenosis. It must be pointed out that a coarse faceted geometry hinders refining the simulation mesh in that critical region, and prevents generating the grid necessary to accurately calculate thin boundary layers. This factor may become important when those layers separate from the artery surface under the action of adverse pressure gradients.

**Fig. 2:**
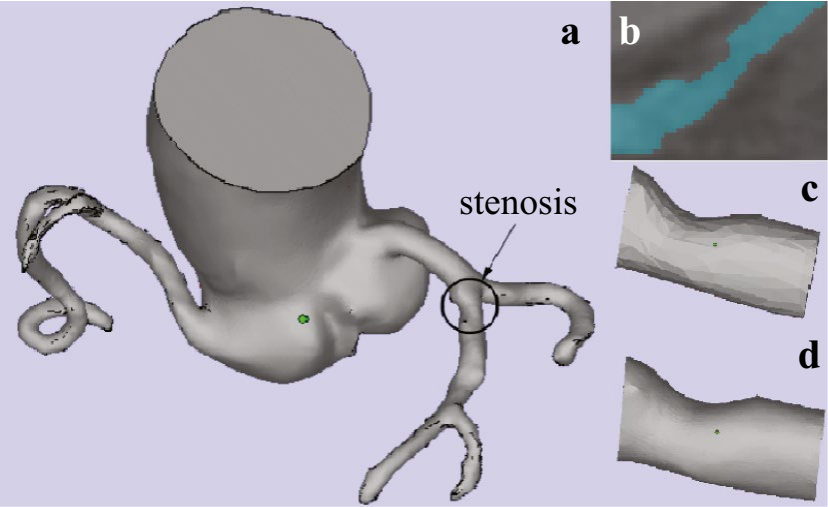
**a** Coronary vasculature determined from segmentation of CTA images. **b** Segmentation of an artery section containing that stenosis. (c y d) Representation of the stenosis surface with 1034 (**c**) 12412 triangles (**d**). The upper image corresponds to the maximum resolution available when the entire fluid domain is considered. The CTA images were provided by the *Hospital Infanta Cristina* in Badajoz (Spain). They were obtained from a 64 detectors scanner (LightSpeed VCT, General Electric Medical Systems, Milwauke, WI, USA) using the retrospective cardiac reconstruction method (SnapShot Segment Mode) with the following acquisition parameters: slice thickness 0.625 mm, rotation time 0.4 s, tube voltaje 120 kV, collimation 0.625 mm and a pitch of 0.18, 0.20, 0.23 or 0.26 (automatically set by the software depending on the patient’s heart rate)

As explained above, the reconstruction of coronary arteries may constitute a significant source of error due to both the image pixel size and the number of triangles used in the geometrical representation. For the sake of illustration, Fig. 2a shows the coronary geometry determined from segmentation of CTA images. As can be observed, the left anterior descending (LAD) artery exhibits a mild stenosis. The image (b) of the figure shows the segmentation of an artery section containing that stenosis. Finally, the images (c) and (d) show the stenosis surface representation with a number of triangles limited by the size of entire fluid domain, and when that restriction is eliminated, respectively. In the present work, we will assess the importance of the stenosis geometry accuracy for the prediction of the FFR value.

In this paper, we will analyze the influence on the pressure drop across a highly-eccentric coronary stenosis of several aspects (approximations) of the simulation method. Specifically, we will consider the accuracy and spatial resolution achieved in reconstructing the stenosis geometry, the velocity distribution at the stenosis inlet, the post-stenotic turbulence, the blood non-Newtonian character, and the values taken by the Reynolds and Womersley numbers. The stenosis geometry will be parameterized by adopting an idealized model that represents a wide class of atheromatous lesions. First, we will simulate the flow through stenoses with varied severities. Then, we will focus on a stenosis with FFR around the critical value 0.8 to determine to what extent the factors mentioned above can influence the outcome of the FFR assessment. We will conclude that this is a geometry-dominated problem, and therefore the limited accuracy and spatial resolution of the stenosis reconstruction is the major source of error in predicting the FFR value. To the best of our knowledge, this aspect of the problem has not been quantitatively analyzed yet.

## Formulation of the problem

**Fig. 3:**
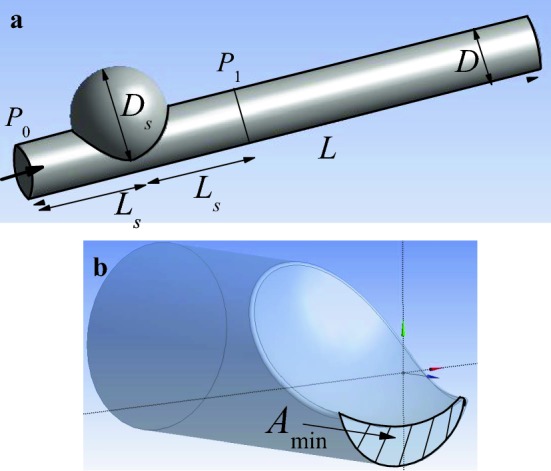
Sketch of the fluid domain considered in our analysis (**a**). Details of the stenosis (**b**)

Consider the flow of a liquid with constant density $\rho$ crossing the domain sketched in Fig. 3a. This domain results from the subtraction of a semisphere of diameter $D_s$ from the cylinder of length *L* and diameter *D*. This subtraction is carried out at a distance $L_s$ from the cylinder inlet. The pre-stenotic $P_0$ and post-stenotic $P_1$ pressures are measured at that distance form the stenosis neck. For fixed values of *L*/*D*, $D_s/D$ and $L_s/D,$ the fluid domain is univocally characterized by the stenosis severity ${\mathcal {S}}=1-4A_{\text{min}}/(\pi D^2),$ where $A_{\text{min}}$ is the minimum cross section area Fig. 3b. This asymmetric stenosis is similar to the real cases described in the “Background” section and considered in, e.g., Refs. [33] and [34].

As mentioned in the “Background” section, the segmentation of the CTA images provides a representation of the real geometry in terms of triangles connected to each other. In order to quantify the quality of that representation, we define the stenosis Surface Resolution Index SRI as $\text{SRI}\equiv N/(S/D^2),$ where *N* is the number of triangles in the stenosis and *S* the area of the surface represented by those triangles. The ratio $S/D^2$ indicates the “complexity” of the surface analyzed: the larger $S/D^2$ the more complex that surface. Therefore, $N/(S/D^2)$ can be regarded as a measure of the surface representation resolution in terms of its complexity. We assign the value $\text{SRI}=\infty$ to the perfectly smooth surface resulting from a mathematical model like the one sketched in Fig. 3.

The liquid viscosity ${\mu}$ is assumed to obey the rheological model

2 $$\begin{aligned} {\mu}={\mu}_{\infty }\left\{ 1+15.2\ \omega \left[ 1+(\lambda \dot{\gamma })^2\right] ^{(n-1)/2}\right\} , \end{aligned}$$

where ${\mu}_{\infty }=0.00350$ Pa s is the viscosity at infinite shear rate, and $\lambda=3.31$ s and $n=0.357$ are constants calculated from experimental data. The blood non-Newtonian character is quantified by the dimensionless number $\omega.$ The values $\omega=0$ and 1 correspond to the Newtonian and Carreau model, respectively.

**Fig. 4:**
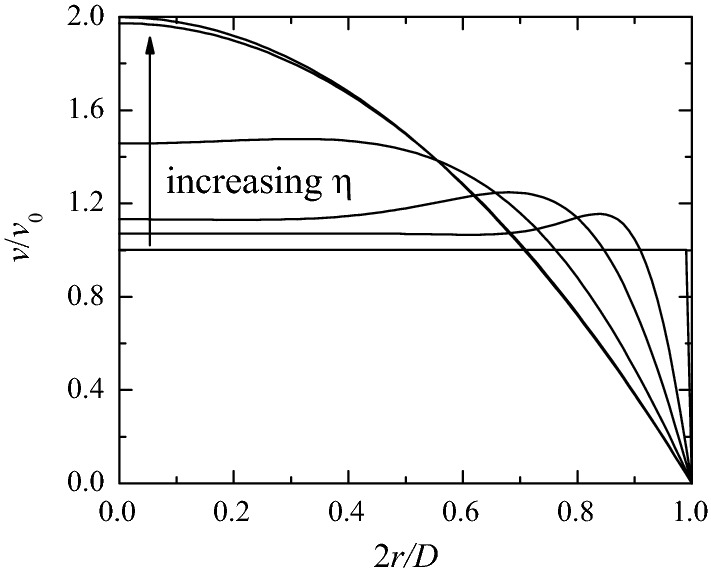
Inlet velocity profile *v* in terms of its mean value $v_{0}$ [Eq. (3)] for $\eta=0,$ 0.05, 0.1, 0.2, 0.5 and 1

The velocity profile at the entrance is perpendicular to that section, and with a magnitude given by the real part of

3 $$\begin{aligned} v(r,t)=& {} v_0(t)\left[ 1-2\eta \, i^{-3/2}\frac{J_1(\eta ^{-1}\, i^{3/2})}{J_0(\eta ^{-1}\, i^{3/2})}\right] ^{-1} \left[ 1-\frac{J_0(\eta ^{-1}\, i^{3/2}2r/D)}{J_0(\eta ^{-1}\, i^{3/2})}\right] , \end{aligned}$$

where *r* is the distance to the cylinder axis, and $v_0(t)$ is the velocity averaged over the inlet section. The velocity distribution (3) is the solution for an infinite pipe subject to a harmonic pressure gradient characterized by a Womersley number value equal to $\eta^{-1}$ [35]. This distribution is considered here just as a family of axisymmetric velocity profiles, where $\eta$ is a dimensionless parameter that measures the deviation from the uniform distribution. In fact, the uniform and parabolic velocity profiles correspond to $\eta=0$ and $\infty,$ respectively (Fig. 4).

**Fig. 5:**
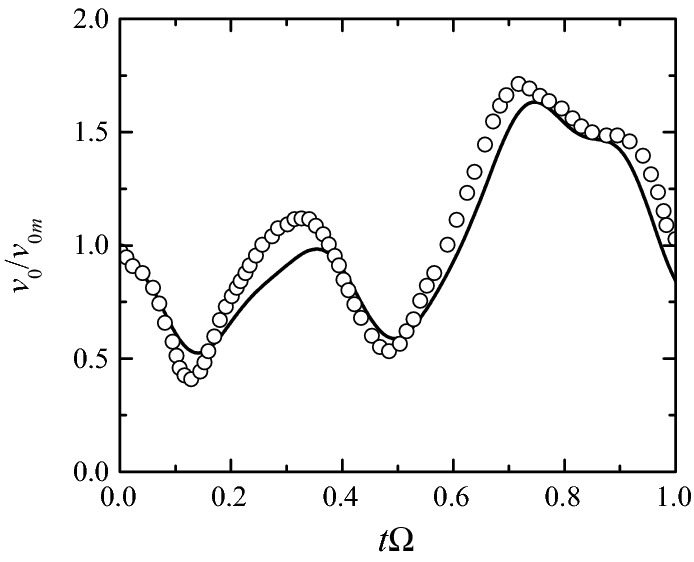
Average velocity $v_0(t)$ in terms of its mean value $v_{0m}$ over the cardiac cycle. The symbols and solid line are the measured values [36] and the approximation using a Fourier series of six terms, respectively

Figure 5 shows the average velocity $v_0(t)$ in terms of its mean value $v_{0m}$ over a cardiac cycle of frequency $\Omega.$ The symbols correspond to experimental data measured in a normal or mildly stenotic coronary artery during hyperaemia [36]. It must be noted that $v_0(t)$ may be significantly affected by the presence of a severe stenosis in the coronary artery. However, there are very few studies where the pressures and velocity in a stenotic coronary artery are simultaneously measured, and those works do not precisely describe the stenosis morphology [37–39]. For this reason, we take the values reported in Fig. 4b of Ref. [36] to simulate the pulsatile flow in our stenosis model (Figs. 13 and 14). In “Influence of the segmentation error on the fractional flow reverse” section, we will examine the effect of the inlet velocity and pressures waveforms on the FFR value by considering waveforms recently measured in the Hospital Infanta Cristina in Badajoz (Spain) when treating a coronary artery with a severe eccentric stenosis. Figure 5 also shows the truncated Fourier series

4 $$\begin{aligned} v_0(t)=v_{0m}+\sum _{n=1}^{n=6} \left[ a_n \sin (2\pi n\Omega t)+b_n \cos (2\pi n\Omega t)\right] \end{aligned}$$

used in the simulations. Typical values for $v_{0m}$ and $\Omega$ are 0.7 m/s and 1.2 s^−1^ respectively.

We will consider $L/D=11.6,$ $D_s/D=1.94,$ and $L_s/D=1.92$ in all our simulations. Once these parameters are fixed, any dimensionless flow quantity is a function of the geometry given by ${\mathcal{S}}$ and SRI, the rheological parameter $\omega,$ the inlet velocity parameter $\eta,$ the Reynolds number $\mathrm{Re}=\rho v_{0m}D/{\mu }_{\infty },$ the Womersley number $\alpha =D(2\pi \Omega \rho /{\mu }_{\infty })^{1/2},$ and the dimensionless time $t^*=t\Omega.$ In particular,

5 $$\begin{aligned} \Pi =\Pi ({\mathcal {S}},\mathrm{SRI},\omega ,\eta ,\mathrm{Re},\alpha ;t^*), \end{aligned}$$

where $\Pi =(P_0-P_1)/\rho v_{0m}^2,$ and $P_0$ and $P_1$ are the reduced pressures averaged over the inlet section and that located at a distance $L_s$ beyond the stenosis, respectively (Fig. 3a). The latter approximately coincides with the distance 2 cm commonly chosen in routine clinical practice. Although the hemodynamic significance of coronary stenoses is essentially assessed in terms of the pressure ratio $P_1/P_0,$ the pressure drop $\Pi$ is the quantity with true hydrodynamic meaning. For this reason, we will examine the function (5) in the first place, and then we will focus on the calculation of FFR.

## Numerical method

In this work, the Navier–Stokes equations were integrated in the incompressible regime with the finite volume method [40] implemented in the commercial software Fluent [41]. Both steady and pulsatile flow simulations were conducted. When turbulence was accounted for, the LES equations were integrated with implicit filtering by calculating the subgrid scale Reynolds stress tensor with the Smagorinsky model. The Smagorinsky coefficient was set to the value 0.13, which leads to accurate predictions in stenosed arteries [22].

The velocity profile (3) was imposed at the inlet section. In the unsteady laminar simulations conducted to calculate the FFR value, we set $\eta=0$ in Eq. (3), which corresponds to the common simplification of uniform flow at the inlet section. The flow was assumed to be fully developed at the outlet section given the large value of *L*/*D*. For this reason, outflow boundary conditions ($\partial a/\partial n=0,$ where *a* is any hydrodynamic quantity and *n* the direction normal to the surface) were prescribed at that section. We verified that this choice leads to virtually the same steady pressure drops as those calculated for constant outlet pressure. On the other side, the non-slip boundary condition was prescribed at the artery inner surface. In the LES simulations, the turbulence intensity was set to zero at the inlet section.

**Fig. 6:**
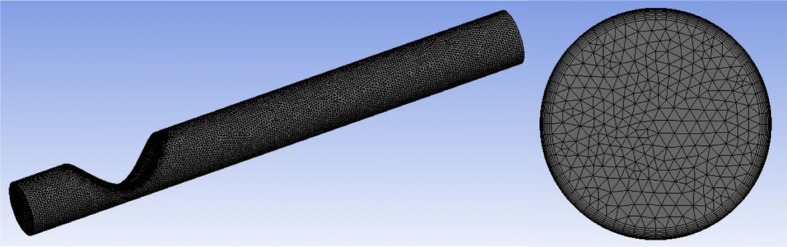
Details of the grid in the laminar case

Use was made of hybrid conformal grids consisting of a structured part formed by rectangular parallelepipeds next to the wall, and an inner unstructured portion formed by tetrahedrons (Fig. 6). The structured part of the grid allows calculating with accuracy the boundary layer in that region. In fact, all the $y^+$ values fell into the interval $0<y^+<0.5.$ The unstructured portion were built by the Automatic mesh algorithm [41]. The number of cells was set to around $3.8\times 10^{5}$ and $1.2\times 10^{6}$ for the laminar and LES simulations, respectively. We verified that the drop of pressure across the stenosis changed in less than 1% when the number of cells was doubled while keeping the same spatial distribution.

The numerical integration of the hydrodynamic equations was carried out with the pressure-based solver. The gradients in the cell center and faces were evaluated with the green-Gauss node-based scheme and a multidimensional Taylor expansion, respectively. The spatial discretization of the pressure equation was conducted with the second-order approximation, while the momentum equations were discretized with the second-order upwind scheme. The velocity-pressure coupling was conducted with the SIMPLE procedure. In the unsteady laminar simulations, the time step $\Delta t^*=0.005$ was much smaller than the period of the cardiac cycle. In the LES simulations, the time step was reduced to $\Delta t^*=10^{-4}.$ The Courant number $C=v\Delta t /\Delta x$ based on the cell size $\Delta x,$ the velocity *v* in the cell, and the time step $\Delta t^*,$ took values in the range 0.1–10. The average value was around 0.6. To check the solution convergence, the pressure drop across the stenosis was monitored in the course of the simulation. In order to reach the quasi-periodic regime in the laminar unsteady case, the simulation was run over three cardiac cycles. The simulation results presented in “Results and discussion” section correspond to the last cycle. The turbulent simulations were run until the flow became statistically steady.

## Results and discussion

### Pressure drop across the stenosis

**Fig. 7:**
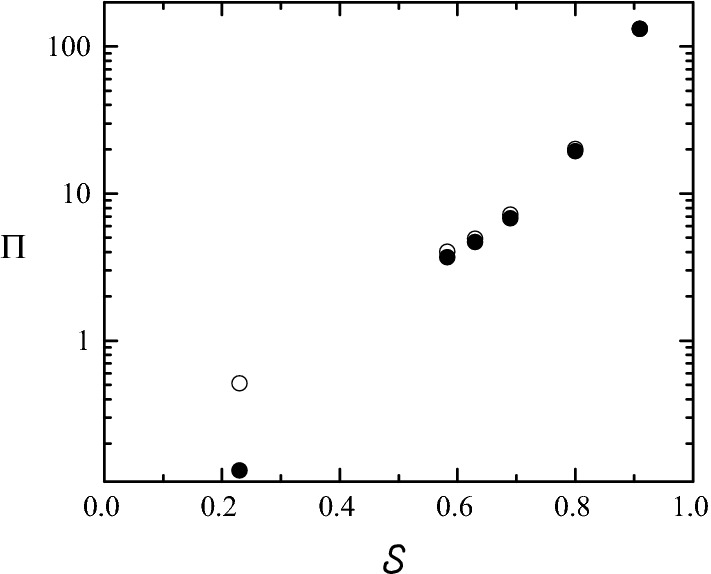
$\Pi$ as a function of ${\mathcal {S}}$ for $\eta=0$ (open symbols) and $\infty$ (solid symbols). The values of the rest of parameters are SRI = $\infty,$ $\omega=0,$ Re = 1268 and $\alpha=0$

**Fig. 8:**
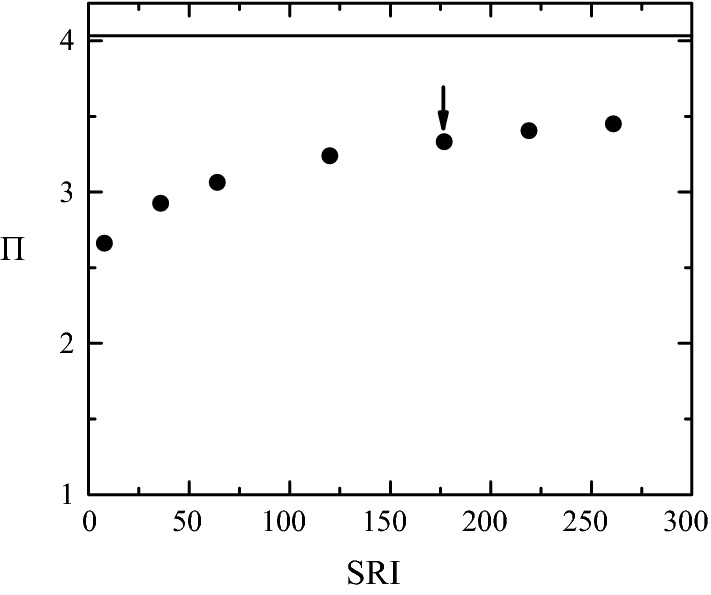
$\Pi$ as a function of SRI. The SRI value of the point marked with an arrow coincides with that of the stenosis shown in Fig. 2c. The solid line corresponds to $\text{SRI}=\infty.$ The values of the rest of parameters are ${\mathcal {S}}=0.583,$ $\omega=\eta =0,$ Re = 1268 and $\alpha=0$

**Fig. 9:**
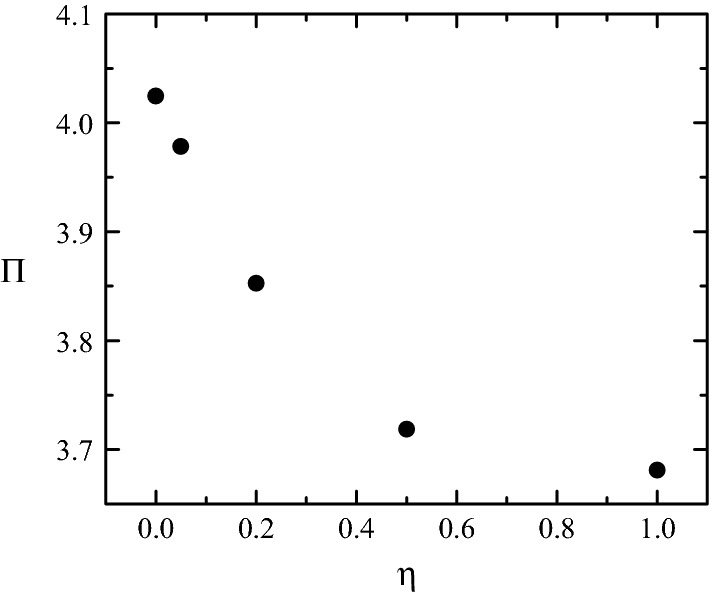
$\Pi$ as a function of $\eta.$ The values of the rest of parameters are ${\mathcal {S}}=0.583,$ $\text{SRI}=\infty,$ $\omega=0,$ Re = 1268 and $\alpha=0$

**Fig. 10:**
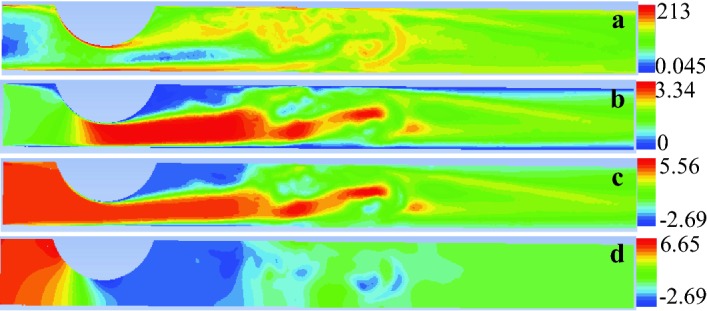
Instantaneous values of vorticity (**a**), velocity (**b**), stagnation pressure difference $P_s-P_{s1}$ (**c**) and static pressure difference $P-P_1$ (**d**) fields in the artery mid-plane. Here, the subscript 1 denotes the average value over the pos-stenotic section defined in Fig. 3a. The results were made dimensionless using *D*, $v_{0m}$ and $\rho v_{0m}^2$ as units of length, velocity and pressure, respectively. In other words, the vorticity, velocity and pressure values were divided by $v_{0m}/D,$ $v_{0m}$ and $\rho v_{0m}^2,$ respectively. The values of the governing parameters are ${\mathcal {S}}=0.583,$  $\text{SRI}=\infty,$ $\omega=\eta =0,$ Re = 1268 and $\alpha=0$

**Fig. 11:**
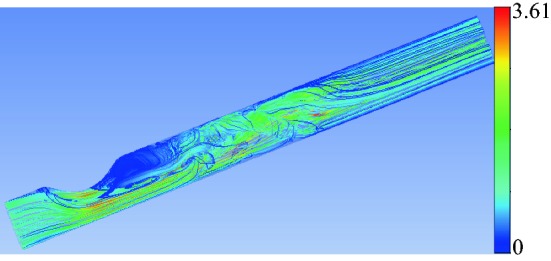
Instantaneous streamlines. The results were made dimensionless using $$v_{0m}$$ as unit of velocity. The values of the governing parameters are $${\mathcal {S}}=0.583,$$$$\text{SRI}=\infty,$$ $$\omega=\eta=0,$$ Re = 1268 and $$\alpha=0$$

**Fig. 12:**
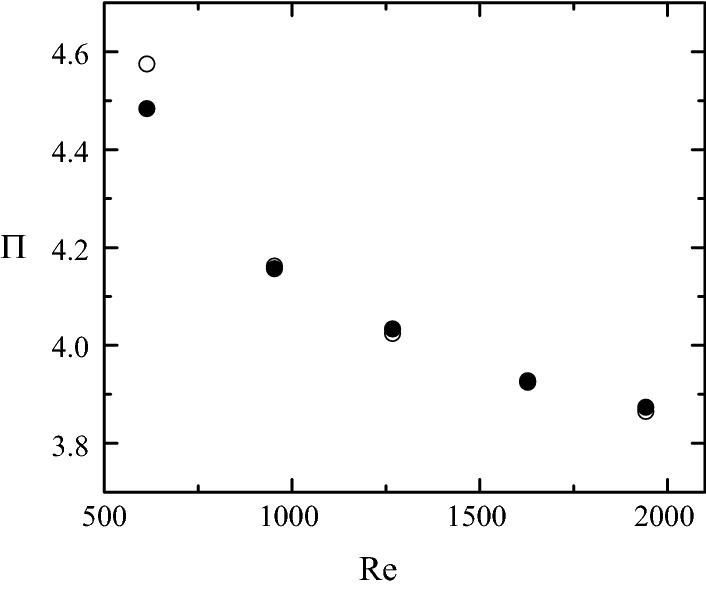
$\Pi$ as a function of Re for $\omega=0$ (solid symbols) and $\omega=1$ (open symbols). The values of the rest of parameters are ${\mathcal {S}}=0.583,$ $\text{SRI}=\infty,$ $\eta=0$ and $\alpha =0$

**Fig. 13:**
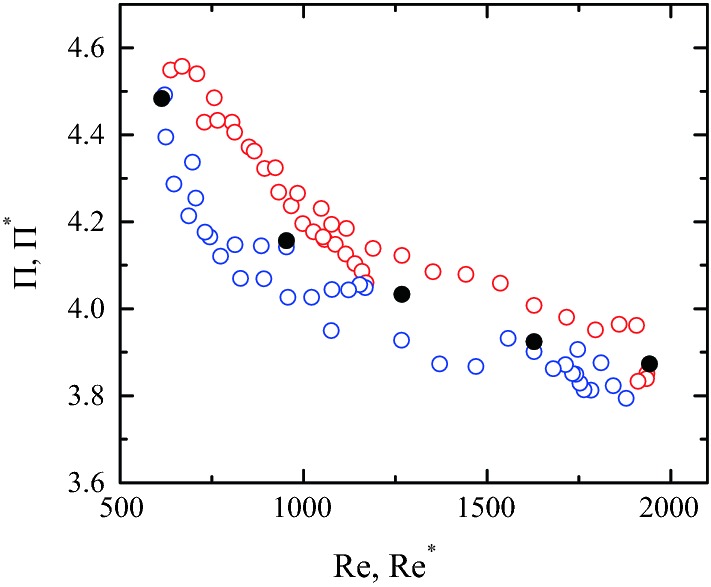
$\Pi (\text{Re})$ and $\Pi ^*(\text{Re}^*)$ for $\alpha=0$ (steady flow, solid symbols) and $\alpha=7.78$ (pulsatile flow, open symbols), respectively. In the pulsatile flow, the red (blue) symbols correspond to the acceleration (deceleration) phase of $v_0(t).$ The values of the rest of parameters are ${\mathcal {S}}=0.583,$ $\text{SRI}=\infty$ and $\omega=\eta=0$

In this subsection, we aim at analyzing systematically the influence of all the simulation aspects described in the “Background” section on the pressure drop across our stenosis model. To keep the computing time at a reasonable level, we conducted this analysis in the steady regime $\left(v_0(t)=v_{0m}\right),\;\alpha=0).$ As will be seen, the results are very similar to those of the pulsatile flow. Figures 7, 8, 9, 10 and 11 show the influence on $\Pi$ of ${\mathcal {S}},$ SRI, $\eta$ hyperaemia and the post-stenotic turbulence for Re = 1268, a typical value in a coronary artery during [36, 42]. Figure 12 shows the dependence of the pressure drop across the stenosis upon the Reynolds number with and without rheological effects. Finally, we analyze the influence of the flow pulsatility for the Womersley number $\alpha =7.78$ (Fig. 13), which also corresponds to a typical value on hyperaemia conditions.

Figure 7 shows the values of $\Pi$ calculated for different stenosis severities. There is a strong influence of this parameter on the pressure drop for severities around 0.6. As will be seen in “Influence of the segmentation error on the fractional flow reverse” section, this value approximately corresponds to the threshold FFR = 0.8 for clinical intervention when the inlet velocity waveform in Fig. 5 is assumed. Therefore, one expects that small variations of the stenosis shape may lead to different outcomes of the FFR assessment. The results presented in Fig. 7 allows one to estimate the errors in $\Pi$ due to the image segmentation process for our stenosis geometrical model. Consider a coronary artery of diameter *D* with a stenosis like that represented in Fig. 3. Assume that the image segmentation produces an error $\delta D_s$ when determining $D_s$ from CTA. That error translates into a variation of the area $A_{\mathrm{min}},$ and therefore of the severity degree ${\mathcal {S}}.$ The resulting deviation of the pressure drop $\Pi$ from its true value can be calculated from the curve $\Pi ({\mathcal {S}}).$ As an example, consider the following values: $D=5.15$ mm (estimated from Fig. 2), $D_s=10$ mm, ${\mathcal {S}}=0.583$ and $\delta D_s$ = + 300 μm (the positive/negative sign stands for an increase/decrease of $D_s$). The latter corresponds to 1-pixel size in Fig. 2b. Due to the error $\delta D_s,$ the stenosis severity and corresponding pressure drop increase from ($S=0.583,$ $\Pi =4.03$) up to $(S=0.63,\;\Pi =4.93).$ To get variations of $\Pi$ smaller than 5%, the segmentation error should be smaller than 100 μm, around one third of 1-pixel size in Fig. 2b. This example shows the importance of accurate image segmentation in the analysis of highly-eccentric stenosis with FFR close to the critical value.

As mentioned in the “Background” section, the surface of a real stenosis is represented in a Fluent simulation in terms of triangles connected to each other, and is stored in the so-called STL format [41]. Another potential source of error in calculating the FFR value is the limited resolution of this faceted geometry. To analyze this aspect of the problem, we first considered the perfectly smooth surface resulting from the mathematical model sketched in Fig. 3. We assign the surface resolution index $\text{SRI}=\infty$ to this surface. Then, several faceted surfaces consisting of different numbers of triangles were generated from the mathematical model. This process was automatically conducted by Fluent when exporting the smooth geometry to the STL format. Finally, we calculated the pressure drop $\Pi$ and the corresponding SRI value for each of those surfaces. It must be noted that the results may significantly depend not only on the SRI value but also on the specific method used to build the faceted geometry. Figure 8 shows $\Pi$ as a function of SRI for the characteristic case ${\mathcal {S}}=0.583.$ The SRI value of the point marked with an arrow coincides with that of the stenosis shown in Fig. 2c, and therefore it can be regarded as a realistic value. The maximum value of SRI corresponds to the maximum number of triangles that can be generated from the procedure described above. The horizontal line indicates the value of $\Pi$ obtained for $\text{SRI}=\infty.$ The pressure drop of the marked point deviates around 19% from that value. Therefore, this aspect of the simulation may constitute a considerable source of error as well.

Figure 7 shows the pressure drops for the uniform $(\eta=0)$ and parabolic ($\eta=\infty$) inlet velocity distributions considering different stenosis degrees ${\mathcal {S}}.$ As can be observed, the uniform and parabolic profiles lead to significantly different results for small ${\mathcal {S}}.$ In fact, the growth of the boundary layer for the plug flow case causes an extra loss of pressure of order unity, and therefore increases the value of $\Pi$ with respect to that of the parabolic profile. This effect was predicted analytically in Ref. [29]. For higher values of ${\mathcal {S}},$ the drop of pressure takes place essentially in the stenotic region, where the flow loses memory of the inlet velocity distribution. For this reason, the values of $\Pi$ obtained in the two cases practically coincide. It must be noted that the numerical simulations provide useful clinical information for our stenosis model when the severity is about 60%, because this case corresponds to $\text{FFR}\simeq$ 0.8 (using the inlet velocity waveform in Fig. 5), and therefore the need of revascularization would be debatable. As can be seen, the pre-stenotic velocity distribution does not play a significant role in this case. Figure 9 shows the slight influence of the inlet velocity distribution for ${\mathcal {S}}=0.583.$

The stenosis eccentricity enhances turbulent motion in the post-stenotic region [21]. It is natural to wonder whether turbulence affects the pressure drop across the stenosis for the Reynolds numbers typically found in the coronary arteries. To answer this question, we have solved the LES model for the average Reynolds number Re = 1268. Figure 10 shows the instantaneous vorticity field measured in the artery symmetry plane. Vorticity vanishes at the entrance because the uniform velocity profile is imposed in that section. It takes small values in the nearly-irrotational core of both the stream in front of the stenosis and the post-stenotic jet. High vorticity values are confined within the thin boundary layer attached to the wall in the pre-stenotic region. The boundary layer separation taking place in the stenosis neck triggers the turbulent motion. The turbulent domain occupies a significant portion of the artery in the post-stenotic region. Relaminarization takes place relatively far away from the outlet. Turbulence slightly enhances energy dissipation. In fact, $\Pi$ increases only in about 3% with respect to the laminar case. As will be explained above, increments of $\Pi$ of this order of magnitude translate into negligible increases of the FFR value. Therefore, the laminar approximation is fully justified when it comes to the calculation of the FFR value for our stenosis model.

Figure 10 also shows the complex flow pattern behind the stenosis. The blood jet originated from the stenosis neck destabilizes, rotates and slows down in the post-stenotic region where the flow reattaches to the artery wall (Fig. 11). That blood deceleration is associated with a significant recovery of static pressure in a relatively short distance. This implies that the FFR value measured in this configuration may considerably depend on the section where the post-stenotic pressure is measured if that section is not located sufficiently far away from the stenosis.

Figure 12 shows the dependency of the pressure drop with respect to the Reynolds number within the range found over the cardiac cycle in a coronary artery stenosis during hyperaemia [36, 42]. As expected, $\Pi$ decreases as Re increases, and reaches an almost constant value for the maximum Reynolds number. This decrease is much smaller than that predicted by the Poiseuille law $\Pi \propto \mathrm{Re}^{-1}$ for a cylindrical shape, which suggests that energy essentially dissipates in the stenotic region due to a process almost independent of the Reynolds number. The Carreau model predicts slightly larger pressure drops for small Re because the viscosity values are significantly larger than ${\mu }_{\infty }$ in this case. The difference between the two constitutive relationships vanishes as the Reynolds number increases owing to the increase of the shear rates. One concludes that the Newtonian approximation holds for our stenosis model.

In order to analyze the influence of the Womerslay number $\alpha$ on the pressure drop across the stenosis, we compared the results for the pulsatile flow with those of the steady regime for the same Reynolds numbers. For this purpose, we defined the Reynolds number $\text{Re}^*=\rho v_{0}(t) D/{\mu }_{\infty }$ and pressure drop $\Pi^*=[P_0(t)-P_1(t)]/\rho v_{0}^2(t),$ which are the instantaneous counterparts of the corresponding dimensionless numbers in the steady regime. Figure 13 shows the comparison between the curves $\Pi^*(\mathrm{Re}^*)$ and $\Pi(\text{Re}).$ The results indicate that $\Pi^*$ varies over the cardiac cycle due to both the variation of $\text{Re}^*$ and the existence of acceleration and deceleration phases. The pressure drop takes larger values in the acceleration phase due to the extra force exerted to increasing liquid momentum, while the opposite occurs in the deceleration phase. The values for the steady regime approximately coincide with those of the pulsatile flow at the Reynolds numbers corresponding to zero acceleration at the inlet section $(dv_0/dt=0).$ The pulsatile character of the flow produces variations of around 5% in the pressure loss across the stenosis with respect to the corresponding steady regime value.

Figures 7, 8, 9, 10, 11, 12 and 13 show the influence of varied simulation aspects on the pressure drop across the stenosis. However, FFR is calculated in terms of the ratio between the pre-stenotic $P_0(t)$ and post-stenotic $P_1(t)$ pressures averaged over the cardiac cycle. For this reason, it is interesting to know how variations of the (instantaneous) pressure drop $\Pi^*(t)$ are translated into variations of the (instantaneous) ratio ${\mathcal {R}}(t)=P_1(t)/P_0(t).$ The pressure ratio ${\mathcal {R}}$ can be obtained from the pressure drop $\Pi ^*$ as

6 $$\begin{aligned} {\mathcal {R}}=\frac{\beta }{\beta -\Pi ^*}, \end{aligned}$$

where $\beta =P_0(t)/\rho v_{0}(t)^{2}$ is the ratio of the static to dynamic pressure proximal to the stenosis. A Taylor expansion shows that a small relative variation $\epsilon _{\Pi ^*}$ of $\Pi ^*$ leads to a relative variation $\epsilon_{\mathcal {R}}$ of ${\mathcal {R}}$ given by the expression

7 $$\begin{aligned} \epsilon _{\mathcal {R}}=\frac{\epsilon _{\Pi ^*}\beta \Pi ^*}{{\mathcal {R}}(\beta -\Pi ^*)^2}+{\mathcal {O}}(\epsilon _{\Pi ^*}^2). \end{aligned}$$

The ratio $\beta$ of the static to dynamic pressure proximal to the stenosis takes values of the order of $10^2.$ In our stenosis model with ${\mathcal {S}}=0.583,$ $\Pi ^{*}\sim 4$ (Fig. 13) and ${\mathcal {R}}\sim 1$ over the cardiac cycle . Using these orders of magnitude in (7), one gets the estimation $\epsilon _{\mathcal {R}}\sim \epsilon _{\Pi ^*}\ \Pi ^*/\beta.$ This analysis shows that errors made in the calculation of $\Pi ^*$ translate into much smaller errors of ${\mathcal {R}}$ (and therefore of FFR) because the dynamic pressure proximal to the stenosis is much smaller than the static one.

The results presented in the previous section and the order-of-magnitude analysis described above allow one to conclude that errors in the calculation of FFR made when assuming a certain velocity profile in front of the stenosis, as well as those associated with the Newtonian and laminar approximations, must be smaller than 1% for our stenosis model with ${\mathcal {S}}=0.583$ (which corresponds to $\text{FFR}\simeq 0.8$ if the inlet velocity waveform in Fig. 5 is assumed). On the contrary, errors in the segmentation of the CTA images and the surface representation may translate into considerable deviations of the FFR value.

### Influence of the segmentation error on the fractional flow reverse

In order to show the validity of the above conclusions, we quantified the influence on the FFR value of one of the two major sources of error: the segmentation of the CTA images. Specifically, we simulated the pulsatile flow across the nominal and distorted geometries considered in “Pressure drop across the stenosis” section. In all the cases, we took $D=5.15$ mm and $D_s=10$ mm. The nominal geometry and distorted geometries correspond to $\delta D_s=0$ and ± 300 μm, respectively. The latter value corresponds to 1-pixel size in Fig. 2b. The severity of the nominal geometry is 58.3%, while ${\mathcal {S}}$ = 63 and 53% for $\delta D_s$ = + 300 and − 300 μm, respectively.

The simulations were conducted by imposing velocity inlet and outflow boundary conditions. In this case, the simulation calculates pressures relative to that of a reference point of the numerical domain. In order to obtain absolute values of the pressure field, the value of this quantity was prescribed at the inlet section. We calculated the FFR values by considering two inlet conditions: (i) the pre-stenotic velocity and pressure waveforms taken from Ref. [36], measured in a mildly stenotic coronary artery during hyperaemia, and (ii) the waveforms measured also on hyperaemia conditions when treating a coronary artery with a severe eccentric stenosis in the Hospital Infanta Cristina in Badajoz (Spain).

**Fig. 14:**
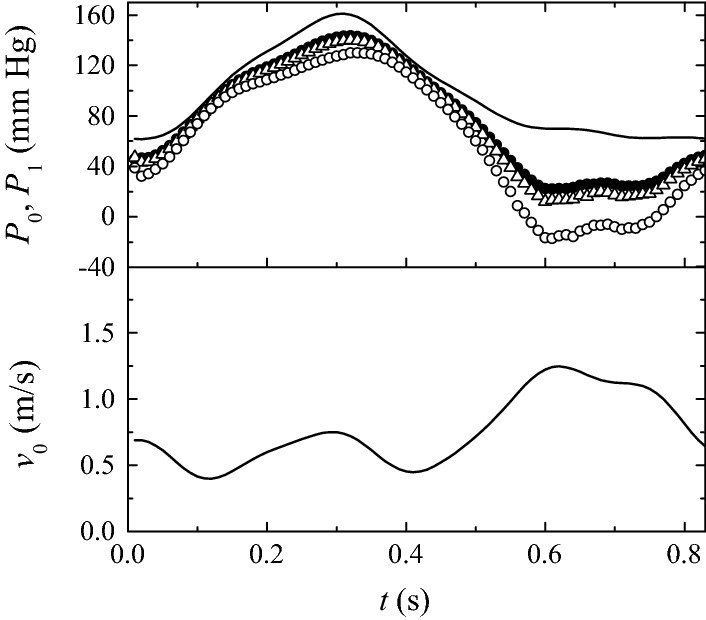
(Upper graph) $P_0(t)$ (solid line) and $P_1(t)$ for $\delta D_s=0$ (solid circles), + 30 μm (triangles) and + 300 μm (open circles). (Lower graph) average velocity $v_0(t)$ at the inlet section. The values of the rest of parameters are ${\mathcal {S}}=0.583,$ $\text{SRI}=\infty,$ $\omega =\eta =0$ and $\alpha =7.78$

Figure 14 shows the inlet velocity (lower graph) and pressure (upper graph) waves for the first of the two cases mentioned above (the inlet velocity is that shown in Fig. 5 too). The post-stenotic pressure $P_1(t)$ was calculated for both the nominal and distorted geometries. As can be observed, the pressure drop $P_0(t)-P_1(t)$ increases/decreases in the time intervals where the velocity increases/decreases. The pressure difference increases for the distorted geometry due to the increase of the stenosis severity, especially in the time interval corresponding to higher velocities. As done in routine clinical practice, we determined the FFR value as $\overline{P}_1/\overline{P}_0,$ where $\overline{P}_0$ and $\overline{P}_1$ are calculated by applying the formula (1). The FFR values obtained from the pressure waves in Fig. 14 are 0.79 and 0.61 for the nominal geometry and $\delta D_s$ = + 300 μm, respectively. Revascularization is not typically conducted for lesions with FFR values very close to 0.8. Therefore, a systematic error of 1 pixel in the image segmentation ($\delta D_s$ = + 300 μm) would modify the clinical decision made from this analysis. Optical coherence tomography (OCT) provides images of the inner wall of a coronary artery with spatial resolutions around 30 μm [43, 44]. Figure 14 also shows the pressure wave calculated with that error. The FFR value obtained from that pressure wave is 0.745, which differs in less than 6% from the true value.

**Fig. 15:**
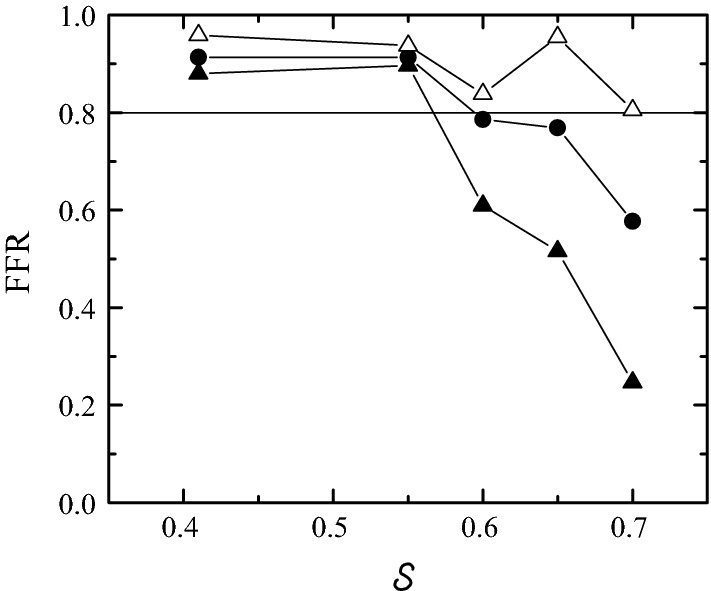
FFR value as a function ${\mathcal {S}}$ for $\delta D_s=0$ (solid circles), + 300 μm (solid triangles) and − 300 μm (open triangles). The values of the rest of parameters are $\text{SRI}=\infty,$ $\omega =\eta =0$ and $\alpha =7.78.$ The horizontal line indicates the critical value ${\mathcal {S}}=0.8.$ The inlet velocity and pressure in the calculations were those shown in Fig. 14

Figure 15 shows the FFR value as a function of the severity ${\mathcal {S}}$ for the nominal geometry and $\delta D_s$ = ± 300 μm. The inlet velocity and pressure in these calculations were the same as those used above, i.e., the pre-stenotic velocity and pressure waveforms taken from Ref. [36]. The FFR value increases for $\delta D_s$ = + 300 due to the increase of the stenosis severity, while the opposite occurs for $\delta D_s$ = − 300 μm. As expected, the deviation between the values for the nominal and distorted geometries increases with the stenosis severity. For $0.575\lesssim {\mathcal {S}}\lesssim 0.7,$ the clinical decision would be modified by 1-pixel error in the image segmentation. The fact that FFR does not decrease monotonously as ${\mathcal {S}}$ increases for $\delta D_s$ = − 300 μm may be attributed to the proximity to the stenosis of the section where $P_1(t)$ is measured.

**Fig. 16:**
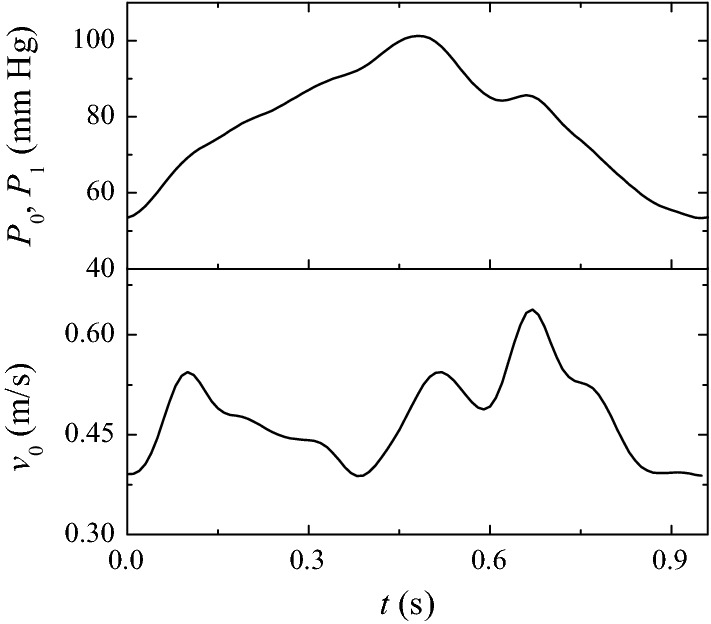
$P_0(t)$ (upper graph) and average velocity $v_0(t)$ (lower graph) at the inlet section measured in the *Hospital Infanta Cristina* in Badajoz (Spain) when treating a coronary artery with a severe eccentric stenosis

**Fig. 17:**
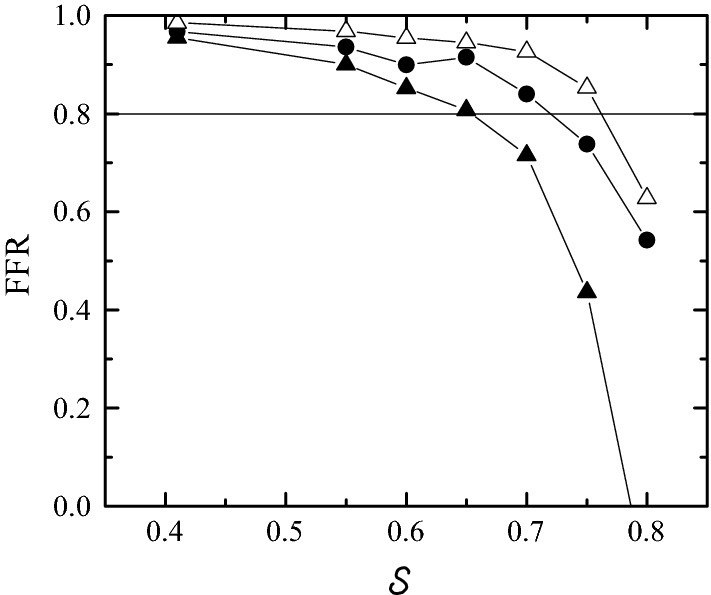
FFR value as a function ${\mathcal {S}}$ for $\delta D_s=0$ (solid circles), + 300 μm (solid triangles) and − 300 μm (open triangles). The values of the rest of parameters are SRI = $\infty,$ $\omega =\eta =0$ and $\alpha =7.78.$ The horizontal line indicates the critical value ${\mathcal {S}}=0.8.$ The inlet velocity and pressure in the calculations were those shown in Fig. 16

As can be observed, a stenosis with a severity of just 58% leads to a FFR value below the threshold 0.8. This result contrasts with the common assumption that the critical FFR value is obtained for severities around 75%. This discrepancy can be attributed to the fact that the velocity wave used to calculate the FFR values in Fig. 15 is that of a healthy or mildly stenotic artery [36], and therefore its magnitude is probably overestimated for severe stenoses. In order to obtain more realistic results for large severities, we have re-calculated the FFR values using the velocity and pressure waveforms recently measured in the Hospital Infanta Cristina in Badajoz (Spain) when treating a coronary artery with a severe eccentric stenosis (Fig. 16). As can be observed, these velocities are significantly smaller than those in Fig. 5. The corresponding FFR results are plotted in Fig. 17. As expected, the FFR values are considerably larger than those in Fig. 15. In fact, the critical severity when $\delta D_s=0$ increases up to around 72% due to the smaller inlet velocities. As can be seen, there is still a significant effect of the image segmentation error on the FFR values.

**Fig. 18:**
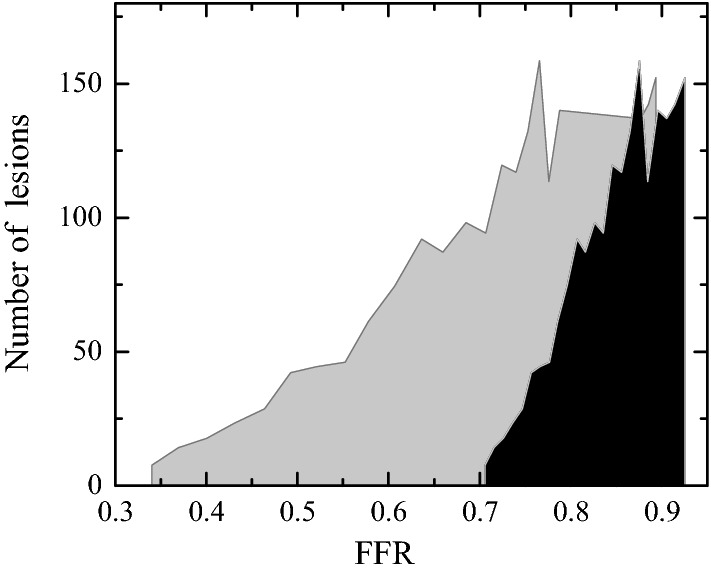
Histogram shown in Fig. 1 of Ref. [45] (dark grey), and the one modified following the procedure described in the text (light grey)

In order to illustrate the potential clinical relevance of the segmentation error, we have correlated our results with a statistical analysis recently published from direct FFR measurements in more than 3000 vessels [45]. The idea is to imagine that the FFR values presented in the histogram in Fig. 1 of Ref. [45] (the dark grey curve in Fig. 18) were calculated from CFD simulations with the right geometry $(\delta D_s=0),$ instead of experimentally. Consider a given FFR value of that histogram. We determine approximately the corresponding stenosis severity from the results shown in Fig. 17 for $\delta D_s=0$ (interpolation is used in the calculations if necessary). We calculate the FFR value for that severity but taking the results in Fig. 17 for $\delta D_s$ = + 300 μm. This new FFR value corresponds to the one that we would have obtained from our CFD simulations if a 1-pixel error had been made in the image segmentation. We take the number of patients in the histogram for the original FFR value, and assign it the to the wrong FFR value. This process is repeated for all the histogram FFR values within the interval $0.7\le \mathrm{FFR}\le 0.9.$ In this way, the original histogram is modified according to the image segmentation error. Figure 18 shows the original and modified histograms. As can be seen, the latter significantly moves towards smaller FFR values. In fact, the number of FFR-guided coronary revascularizations $(\text{FFR}<0.8)$ would pass from 360 to 1153 out of the 1944 cases within the interval $0.7\le \text{FFR}\le 0.9.$

Based on our analysis, one can propose a two-step method to improve the CFD calculation of the FFR value for highly-eccentric coronary stenoses. In the first step, one determines the internal geometry of the large-scale numerical domain (including the aorta and the rest of peripheral vasculature) from CTA images of the cardiac vasculature, and conduct the corresponding numerical simulation. This calculation allows one to determine both the time-dependent velocity profile and pressure waveform at a given section of the pre-stenotic segment. If possible, that velocity profile might be re-calculated by scaling all the velocities so that the flow rate coincides with that measured from any relatively non-invasive technique, like MR phase-contrast flow measurements [27, 28]. In the second step, images of the stenosed coronary artery are acquired to increasing the accuracy of the image segmentation and the resolution of the surface representation in that region. For this purpose, OCT might be used [43, 44]. Then, the FFR value is determined by simulating the pulsatile flow across that region using the inlet pressure and velocity obtained in the first step. A similar idea was proposed in Ref. [19]. In that case, the pre-simulation was conducted to calculate just the pre-stenotic pressure, and the uniform velocity profile was assumed in that region.

## Conclusions

FFR-guided revascularization offers important advantages over other assessments such as CTA or AME. For instance, it proves to be long-lived and cost-saving, exhibits lower rates of major adverse cardiac events, and correctly classifies as hemodynamically insignificant many lesions judged “obstructive” by CTA or AME. However, FFR assessment requires the catheterization of the coronary arteries to measure the pressure waveforms both proximal and distal to the stenosis. CFD has arisen as a useful alternative to this invasive technique [10, 11]. It can predict the FFR value just from CTA images of the patient cardiac vasculature when some approximations are taken. The major concern when conducting CFD simulations is typically the validity of the inlet and outlet boundary conditions necessary to correctly pose the mathematical problem. This difficulty has been addressed by enlarging the numerical domain, so that it ends where certain boundary conditions can be safely imposed [10, 11]. Much less (if any) attention has been paid to the critical role played by the geometrical reconstruction and representation of the stenosis surface. It must be pointed out that the disparity between the scales of the fluid domain considered in the simulations and the critical stenotic region considerably hinders an accurate geometrical reconstruction of the latter. Our results have shown that both the limited resolution of the CTA images and a coarse representation of the artery surface constitute an important obstacle to determine the FFR value of highly-eccentric stenoses with sufficient accuracy. It must be noted that eccentric coronary plaques are prone to dynamic changes in stenosis geometry during the cardiac cycle, especially partial collapse with decreasing distending pressure during higher hyperemic velocity in diastole. These changes in geometry may well exceed the effect of imaging inaccuracy. The spatial resolution of images acquired with optical coherence tomography (OCT) may be sufficient to ensure accurate predictions for the FFR value.

We have extended our study to analyze the influence of a number of approximations on the pressure drop across highly-eccentric coronary stenoses. For this purpose, we have parameterized the stenosis geometry by adopting an idealized model that represents a wide class of stenoses caused by atheromatous lesions. We have considered realistic values of the rest of parameters governing the problem (artery diameter, velocity and pressure at the inlet section,…). The conclusions derived from this analysis are expected to be qualitatively valid for real stenoses in curved, flexible and moving vessels with side branches. Our results on the pressure drop across the stenosis confirm the common belief that the flow can be regarded as Newtonian and laminar. In addition, the inlet velocity distribution plays a secondary role in severe stenoses because of the very localized character of the pressure drop in the artery narrowing. Finally, the flow pulsatility has little influence on the pressure drop due to both the moderate value of the Womerslay number and the smooth temporal evolution over the cardiac cycle of the velocity in the coronary artery inlet.

We have restricted our analysis to the geometry described in “Formulation of the problem” section because the majority of coronary artery stenoses are caused by atheromatous lesions producing an eccentric narrowing similar that geometry [1]. A natural question is whether the above conclusions obtained for highly-eccentric coronary stenoses can also be applied to concentric ones. Most of the features analyzed here are not expected to be specific of eccentric stenoses; in particular, the fact that both the limited resolution of the CTA images and a coarse representation of the artery surface constitute important obstacles to calculate accurately the FFR value. On the contrary, the destabilization of the blood jet associated with a significant adverse pressure gradient may considerably depend on the stenosis eccentricity degree. This is an important phenomenon because it can make the FFR value very sensitive to the location where distal pressure is measured. The sample of analyzed geometries must be enlarged to complete the present study. It is also interesting to assess quantitatively the validity of the above conclusions for real patient-specific geometries and their corresponding hemodynamic conditions.

The present work raises the question of whether the image spatial resolution obtained in standard CTA is high enough for CFD to provide reliable predictions in FFR assessment. To answer this question, one must conduct a systematic study of the effects produced by choices made in the segmentation process on the resulting FFR value. In particular, attention must be paid to the range of values in the Hounsfield scale corresponding to the area delimited by the artery inner wall.

In principle, the FFR value could be also determined by conducting experiments on models fabricated with 3D printing [46, 47]. Increasing the size of the model does not reduce the error coming from image segmentation, but does decrease that associated with a poor representation of the artery surface. In fact, the use of big models allows one to increase the number *N* of triangles of the faceted geometry while keeping the ratio $S/D^2$ constant, which results in higher values of SRI. In this sense, high-resolution printers have proved to be capable of forming precise replicas of big models of coronary arteries [47].
